# Enhanced Landslide Visualization and Trace Identification Using LiDAR-Derived DEM

**DOI:** 10.3390/s25144391

**Published:** 2025-07-14

**Authors:** Jie Lv, Chengzhuo Lu, Minjun Ye, Yuting Long, Wenbing Li, Minglong Yang

**Affiliations:** 1 Department of Earth Science and Technology, City College, Kunming University of Science and Technology, Kunming 650093, China; 2022128525140@stu.kust.edu.cn (Y.L.); liwenbing@stu.kust.edu.cn (W.L.); 20130051@kust.edu.cn (M.Y.); 2 School of Land and Resources Engineering, Kunming University of Science and Technology, Kunming 650093, China; 20232201100@stu.kust.edu.cn (C.L.); yeminjun@stu.kust.edu.cn (M.Y.)

**Keywords:** LiDAR-DEM, image fusion, fractal model, enhanced display, landslide trace

## Abstract

In response to the inability of traditional remote sensing technology to accurately capture the micro-topographic features of landslide surfaces in vegetated areas under complex terrain conditions, this paper proposes a method for enhanced landslide terrain display and trace recognition based on airborne LiDAR technology. Firstly, a high-precision LiDAR-DEM is constructed using preprocessed LiDAR point cloud data, and visual images are generated using visualization methods, including hillshade, slope, openness, and Sky View Factor (SVF). Secondly, pixel-level image fusion methods are applied to the visual images to obtain enhanced display images of the landslide terrain. Finally, a threshold is determined through a fractal model, and the Mean-Shift algorithm is utilized for clustering and denoising to extract landslide traces. The results indicate that employing pixel-level image fusion technology, which combines the advantageous features of multiple terrain visualization images, effectively enhances the display of landslide micro-topography. Moreover, based on the enhanced display images, the fractal model and the Mean-Shift algorithm are applied for denoising to extract landslide traces. Compared to orthophotos, this method can effectively and accurately extract landslide traces. The findings of this study provide valuable references for the enhanced display and trace recognition of landslide terrain in densely vegetated areas within complex mountainous areas, thereby providing technical support for emergency investigations of landslide disasters.

## 1. Introduction

Landslides, as frequent and highly destructive geological disasters, pose a severe threat to both human society and the natural environment. The topographic and geomorphic features of landslide surfaces are important and intuitive manifestations during the processes of landslide movement, deformation, and instability damage [1,2]. Traditional landslide monitoring methods are constrained by factors such as terrain, vegetation cover, and meteorological conditions. For example, dense vegetation may obscure surface traces of landslides, thereby limiting the field of view of remote sensing detection technologies and interfering with the spectral information of optical remote sensing data [3]. Consequently, accurately and comprehensively describing the micro-topographic features of landslide areas is of great significance for understanding the movement and evolutionary process of landslides, grasping their current activity status, and predicting their future activity [4,5].

In recent years, LiDAR (Light Detection and Ranging) technology has significantly improved the accuracy of terrain data acquisition by virtue of its ability to penetrate vegetation to obtain surface data, providing high-resolution Digital Elevation Models (DEMs) [6,7]. Airborne LiDAR technology, with its unparalleled high-resolution topographic modeling capability and vegetation-penetrating performance, has demonstrated distinctive advantages in landslide identification. It has rapidly evolved into an indispensable tool for geohazard monitoring and quantitative risk assessment [8,9]. Jaboyedoff [10] et al. conducted a comprehensive synthesis of LiDAR-derived DEM applications for geological hazards, including landslides, rock avalanches, and debris flows and covering mass-movement detection and characterization, hazard assessment, and susceptibility mapping, which provides a theoretical foundation for subsequent studies. Through comparisons between LiDAR and traditional methods, Görüm et al. [11] demonstrated that the former can identify far more landslides than the original inventory and accurately recognize small-scale landslides, highlighting its critical advantages in complex terrains and forest-covered areas. Chen et al. [12] further validated the effectiveness of LiDAR integrated with feature selection and random forest algorithms in achieving high-precision landslide boundary extraction under dense vegetation cover in the Three Gorges Reservoir area. However, these studies mainly focus on improving the landslide identification capability, lacking attention to the automation level of the identification process. Most of them rely on manual interpretation and empirical parameter setting, which limits the generalizability and stability of the methods.

In the realm of terrain visualization and image enhancement, a multitude of methodologies have been proposed to augment the efficacy of landslide feature recognition. Chen [13] et al. leveraged airborne LiDAR to generate a DEM and successfully identified the location and scale of landslides through hillshade analysis, color-enhanced display, and 3D simulation. Sun [14] adopted a similar approach to identify landslide hazards in Danba County, and field verification results demonstrated a high level of identification accuracy. Guo et al. [15] introduced the Sky View Factor (SVF) method, which eliminated the influence of a single light source on traditional mountain shadows and improved the landslide identification accuracy. Guo et al. [16] employed SVF on this basis to generate quasi-three-dimensional terrain images for interpreting and identifying geological disasters. Field verification showed that it outperformed the classic mountain shadow method. Verbovsek et al. [17] and Han et al. [18] proposed the VAT method, which fuses multi-layer information (including mountain shadows, slope, openness, and SVF), effectively enhancing the visibility of landslide geomorphic features and improving identification accuracy. Although these visualization methods perform well in landslide identification, they mainly rely on static image enhancement, making it difficult for them to cope with weak landslide signals under dynamic changes or complex geomorphologies, and lack an effective integration mechanism for multi-source data.

In addition, LiDAR has been widely used in the dynamic monitoring and evolutionary process analysis of landslides. Pellicani et al. [19] combined LiDAR and UAV remote sensing data, compared the topographic changes before and after a landslide, and revealed the kinematic characteristics and evolutionary process of the Montescaglioso landslide in Italy. Liu et al. [20] constructed a landslide identification model based on multi-temporal LiDAR data and geomorphic parameters. After the exclusion of small landslides, the prediction accuracy reached up to 76.6%, indicating that this method has good applicability in landslide information extraction. Lo et al. [21] combined an SVF-enhanced DEM with terrain slope and elevation maps and used the Analytic Hierarchy Process (AHP) to assess landslide hazard, accurately reflecting the evolution trend of the Oso landslide around 2014. However, current dynamic monitoring research based on LiDAR is still in its preliminary stage. The prediction accuracy of models is limited by the sample size and the completeness of time series, and it is difficult to achieve real-time tracking and intelligent early warning of landslide evolution processes.

Multiple regional case studies have further verified the adaptability and effectiveness of LiDAR in different geographical environments. Wang et al. [22] took the Jiuzhaigou earthquake area as an example, using LiDAR and red stereo map processing methods to overcome the limitations of optical remote sensing in densely vegetated areas, and improved the remote sensing interpretation accuracy of collapses, landslides, and debris flow disasters. Yi [23] used LiDAR to collect point cloud data of the Liziya landslide, generated a high-precision DEM, and successfully identified and extracted the landslide boundary. Pradhan et al. [24] proposed a semi-automatic landslide detection technique based on saliency enhancement. By combining morphological analysis with the fuzzy C-means clustering algorithm, this method enables the automatic identification of landslide traces. These case studies demonstrate the practicality of LiDAR in diverse environments, but most methods still lack universality and standardized procedures, making it difficult to transfer and reuse them across different regions. Although significant achievements have been made in landslide identification in existing studies, there are still problems such as a strong dependence on manual interpretation, a limited ability to identify small-scale landslides, and insufficient multi-source data fusion. Therefore, developing a more intelligent and automated landslide identification framework, especially explorations in image fusion, fractal modeling, and deep learning integration, will become a key direction for improving landslide monitoring accuracy.

In summary, the analysis of the aforementioned studies shows that the utilization of LiDAR technology has predominantly focused on single visualization methods for landslide terrain using high-resolution DEM data, and there have been studies integrating multiple visualization images [17,18,25,26]. However, research on the enhanced display of landslide terrain through the fusion of multiple terrain visualization images and the utilization of enhanced display images for landslide trace recognition remains relatively scarce. Therefore, generating DEMs from post-disaster airborne LiDAR data to achieve enhanced display and recognition extraction of landslide traces is an urgent issue that needs to be addressed.

This study attempts to explore a method for the enhanced display and trace recognition of landslides based on an airborne LiDAR-derived DEM. Taking the landslide on the left side of the Dabaini Debris Flow Gully in Dongchuan District as the research object, high-resolution DEM data generated from airborne LiDAR were utilized to visually express the terrain features of the area through terrain visualization methods like hillshade, SVF, openness, and slope. By integrating a pixel-based image fusion technology, a new method for the enhanced display of landslide terrain was introduced. Furthermore, the study combined Geographic Information System (GIS), fractal geometry, and image processing technologies to achieve the extraction of landslide traces. The integration of these technologies not only improves landslide identification capabilities but also provides researchers in related fields with new perspectives, offering scientific support for geological disaster monitoring and landslide risk assessment.

## 2. Overview of the Study Area

**Figure 1:**
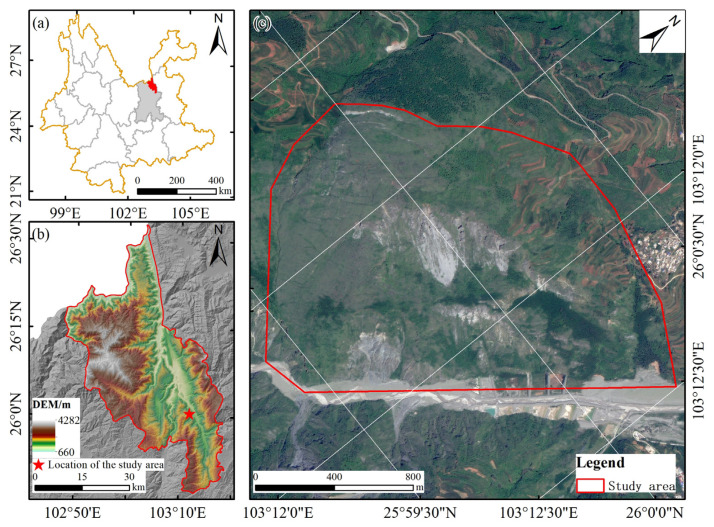
Overview of the study area. (**a**) Yunnan Province: (**b**) Dongchuan District; (**c**) Study Area.

The research area is located in the Xiaobaini River Valley in the middle and lower reaches of the Xiaojiang River Basin in Dongchuan District, Kunming City, Yunnan Province, as illustrated in Figure 1, covering an area of approximately 2.44 km^2^. The average annual temperature in this area is 14.9 °C, and the annual precipitation is 1000.5 mm, concentrated from May to September, with a maximum monthly precipitation of 208.3 mm and a maximum daily precipitation of 1533 mm. The area enjoys 2327.5 h of annual sunshine, with an annual evaporation rate of 1856.4 mm, a maximum wind speed of 40 m/s, and an average annual relative humidity of 76%. Elevations range from 695 m to 4344.1 m, with a total elevation difference of 3649.1 m, and the total area of Dongchuan District is 1674 km^2^. This area is a typical development zone prone to rainstorm-induced debris flows, earning it the nickname “Natural Debris Flow Museum”. Situated at the intersection of the eastern and western branches of the Xiaojing Fault Zone, it has been shaped by tectonic movements, forming high mountains, deep valleys, and steep landforms. Landslides and collapses are widespread, and the complex terrain with prominent gullies provides abundant cases for landslide research, aiding in the study of landslide triggering mechanisms and preventive measures and offering significant reference value for disaster management in regions with similar terrain.

## 3. Data

### 3.1. Data Collection

**Figure 2:**
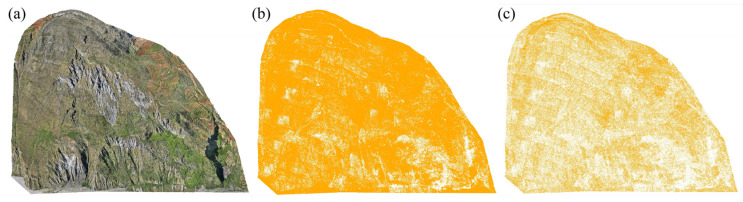
Comparison of point cloud data before and after preprocessing in the study area: (**a**) original point cloud; (**b**) filtered ground points; (**c**) down-sampled ground points.

Data for the study area was collected using a DJI M350RTK UAV (Shenzhen DJI Innovation Technology Co., Ltd., Shenzhen, China) equipped with the DJI Zenmuse L2 (Shenzhen DJI Innovation Technology Co., Ltd., Shenzhen, China) airborne LiDAR system, employing terrain-following flight technology. This technology demonstrated significant adaptability and maneuverability in complex terrain, enabling stable data acquisition at relatively lower altitudes [27]. Data collection was conducted in the experimental area on 15 November 2024, utilizing the CGCS2000 coordinate system with a central meridian of 102 E, and elevations were recorded using geodetic heights. The designed relative flight altitude was 190 m, with a lateral overlap of 30% and a flight speed of 10 m/s. The specifications of the DJI Zenmuse L2 airborne LiDAR system and flight parameters are outlined in Table 1. LiDAR point cloud data with an average point density exceeding 445 pts/m^2^ was obtained, with the original point cloud data illustrated in Figure 2a. Given the steep slopes, significant elevation differences, severe soil and water erosion at the front edge, intense deformation at the rear edge, and high-risk factors associated with the landslide, ground control points were not deployed during data acquisition; instead, data was acquired using a no-control point mode.

### 3.2. Data Preprocessing

**Figure 3:**
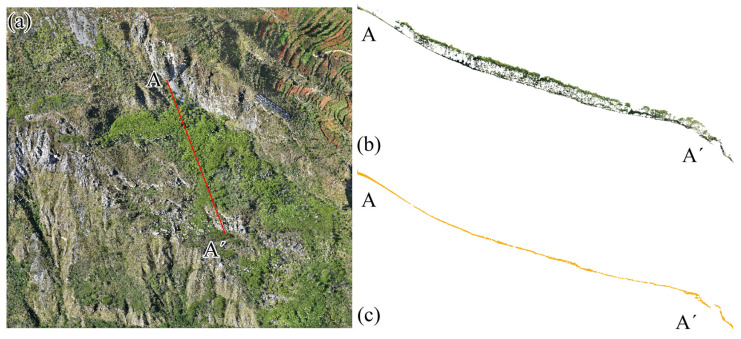
Comparison of profiles before and after point cloud filtering: (**a**) position of AA’ profile; (**b**) profile before filtering; (**c**) profile after filtering.

Data preprocessing employed the strip adjustment and strip stitching functions in the LiDAR360 v2.2 software to address redundancy issues in the initial point cloud data [28]. The point cloud data obtained through strip adjustment and stitching contained noise and outliers in the raw point cloud due to factors such as flying objects (e.g., birds) and collection errors (e.g., multipath errors). These errors were removed using the denoising functionality in the LiDAR360 software. The algorithm searches for a specified number of neighboring points for each point, calculates the average distance from the point to its neighbors, and then computes the median and standard deviation of these average distances. If the average distance exceeds a maximum threshold, the point is deemed noise and removed. Given the high vegetation coverage in certain areas of the study area, the denoised point cloud still contained numerous non-ground points due to vegetation interference, as illustrated in Figure 3a. To extract ground points from the point cloud, a progressive triangulation filtering algorithm in the TerraSolid V024 software was initially applied, followed by manual verification and correction of any misclassified points. Comparisons of filtering profiles are shown in Figure 3b,c. To ensure the accuracy and reliability of the filtering results, a cross-tabulation evaluation system proposed by the International Society for Photogrammetry and Remote Sensing (ISPRS) in 2003 was utilized for quantitative analysis [29]. The analysis revealed an omission error of 1.67%, a misclassification error of 4.84%, and an overall misclassification rate of 2.31% for the point cloud, with the filtering results depicted in Figure 2b. Due to overlaps between flight strips and passes during the point cloud data collection, the point cloud density in these overlapping areas was significantly higher than in other parts. To reduce data redundancy’s impact on subsequent processing, the voxel sampling method was employed to down-sample the ground point cloud data, resulting in a point cloud density of over 26 pts/m^2^, as illustrated in Figure 2c. To verify the measurement accuracy of the point cloud data, real-time kinematic (RTK) surveying technology was utilized to conduct field measurements of prominent ground feature points. By comparing these accurately measured coordinate values with those in the point cloud data, the results show that at a flight altitude of 190 m, both horizontal and vertical accuracies were better than 10 cm, meeting the requirements for landslide identification and assessment tasks.

### 3.3. Construction of LiDAR-DEM

**Figure 4:**
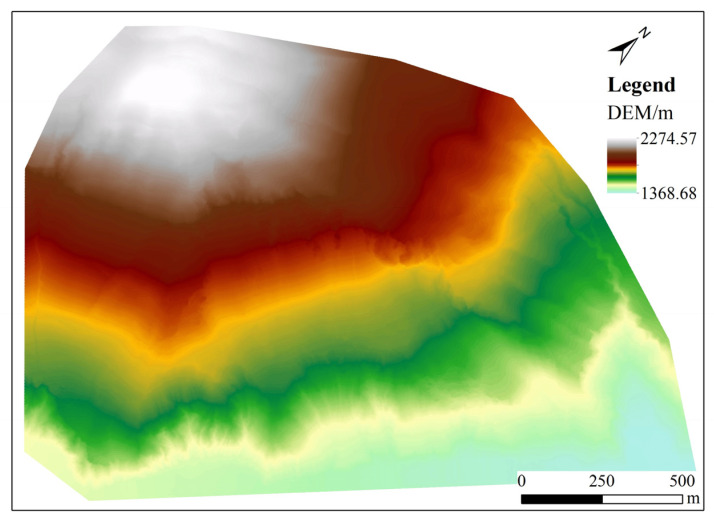
LiDAR-DEM image of the study area.

The factors affecting DEM accuracy mainly include terrain complexity, data processing, point cloud density, and interpolation methods [30]. For DEMs generated by software, the choice of interpolation method has the most significant impact on their accuracy, directly determining the accuracy of the DEM data. Common regular grid interpolation methods include Ordinary Kriging (OK), Trend Surface, Radial Basis Function, Spline, Natural Neighbor, and Inverse Distance Weighting (IDW). In complex terrains, the IDW and OK interpolation algorithms offer the highest accuracy, while the Spline interpolation algorithm may produce distortion effects, and the Trend method is not suitable for interpolation in complex terrains [31]. In this study, the OK interpolation algorithm was employed to construct the DEM for the study area, with the interpolation parameters set as a Gaussian function for the variogram, 12 search points, and an eight-direction search, interpolating the down-sampled ground point data to generate a DEM with a resolution of 0.5 m, as demonstrated in Figure 4.

To ensure the accuracy and reliability of the DEM product, this paper employed a cross-validation method, which divides the ground points into 95% training samples to generate the DEM and 5% validation samples to assess the DEM’s accuracy. Based on the differences between the elevation values of the validation sample points and the interpolated DEM values, three accuracy evaluation metrics were calculated: the Root Mean Square Error (RMSE), Mean Absolute Error (MAE), and Coefficient of Determination (R^2^). Among them, RMSE and MAE are negatively correlated with the accuracy of the interpolated DEM, meaning higher values indicate lower accuracy. Conversely, R^2^ is positively correlated with DEM accuracy, with higher values indicating greater accuracy. Statistical calculations yielded an RMSE of 0.1425 m, an MAE of 0.0958 m, and an R^2^ of 0.9900.

## 4. Methods

**Figure 5:**
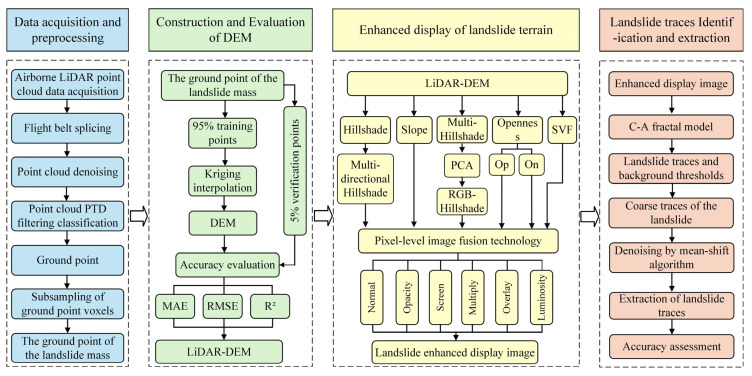
Overall process flowchart.

The process of terrain enhancement display and landslide trace identification using airborne LiDAR technology consists of four steps: (1) data acquisition and preprocessing; (2) construction and accuracy assessment of the LiDAR-derived DEM; (3) pixel-level image fusion-based terrain enhancement for landslide visualization; and (4) extraction of landslide traces using a fractal model based on the enhanced imagery. The technical workflow of this study is illustrated in Figure 5.

### 4.1. LiDAR-DEM Visualization

#### 4.1.1. Hillshade

Hillshade visually represents terrain features by simulating the illumination of the Earth’s surface by sunlight [32]. Areas directly illuminated by the sun appear bright, while shaded areas appear dark, and the interplay of light and shadow creates a three-dimensional effect on a two-dimensional terrain image [33]. The hillshade effect is closely related to the solar azimuth angle (SAA) and the solar elevation angle (SEA), where SAA indicates the angle between the sun and the north direction, ranging from 0° to 360°, while SEA indicates the sun’s height above the horizon, ranging from 0° to 90°. Hillshade images are typically grayscale, with values ranging from 0 to 255, where 0 represents black and 255 represents white.

Although Hillshade effectively reveals terrain features in geological disaster areas, micro-topography is often obscured due to the influence of SAA and local terrain. Therefore, interpreting hillshade images requires combining multiple shadow images from different directions. To reduce the over-bright or over-dark effects caused by different SAA values and improve the recognition of terrain features, three hillshade images with different SAAs can be combined through RGB synthesis to produce a color-displayed image [34]. Images illuminated from multiple angles are complementary, and principal component analysis (PCA) can be employed to optimize the display effect.

#### 4.1.2. Terrain Slope

Terrain slope maps are mainly used to display the steepness of the Earth’s surface, making them particularly suitable for areas with significant elevation changes, while their effectiveness is more limited in flat areas. The calculation principle is based on the DEM, assessing the maximum slope change between each pixel and its surrounding neighboring pixels. In practical calculations, the slope of each pixel is typically determined by considering its slope in relation to its eight neighboring pixels. The slope represents the first derivative of the DEM, representing the maximum rate of change between adjacent units, and is independent of the aspect. The calculation formula is as follows:

(1) $$tan\alpha =\frac{\left(y_{1}-y_{2}\right)}{\left(x_{1}-x_{2}\right)}$$

Slope maps are a commonly used visualization technique that presents a three-dimensional effect by displaying steep slopes through grayscale inversion. However, a single slope map struggles to distinguish between positive and negative terrains due to their similar colors and shading [35]. Therefore, when analyzing terrain, it is necessary to integrate other information to differentiate between terrain types. Additionally, research by Chiba et al. [36] indicates that the human eye is more sensitive to the color red, which helps in identifying subtle geomorphic features. Thus, slope maps displayed in red can also enhance the visualization of terrain details.

#### 4.1.3. Sky View Factor

**Figure 6:**
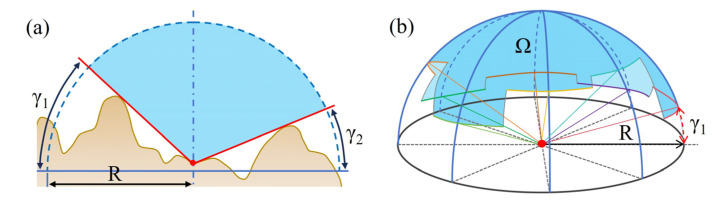
SVF principle (modified according to Zakšek K [37]).

The Sky View Factor (SVF) addresses the shadowing issues caused by a single light source through a diffuse reflection method, quantitatively describing the openness of the terrain surface [36], which is significant for studying urban thermal radiation and the heat island effect [37], and it measures the visibility of the sky from a specific point and is a dimensionless indicator [15]. When calculating SVF, it is assumed that light is uniformly distributed and other light sources are excluded, while also considering the limitations imposed by ground objects on the field of view. The calculation of SVF involves integrating over different elevation angles and accounting for the influence of Earth’s curvature on sky visibility (a). Compared to other terrain visualization techniques, SVF reduces interference from a single light source while preserving the macroscopic features of the terrain, thereby making subtle topographical features clearer (b). The principle of SVF is illustrated in Figure 6.

The calculation formula for SVF [15] is as follows:

(2) $$V=1-\frac{\sum_{i=1}^{n}sin\;\gamma_{i}}{n}$$

where n represents the number of search directions and $\gamma_{i}$ represents the elevation angle for different directions.

The calculation of SVF is influenced by two parameters: horizontal search direction (HSD) and search radius (SR), with values ranging from 0 to 1. When the SVF value is 1, it indicates that the hemisphere above the observation point is completely visible, resulting in a brighter image; when the SVF value is 0, it indicates that there is almost no visible sky above the observation point, and the image appears darker.

#### 4.1.4. Terrain Openness

**Figure 7:**
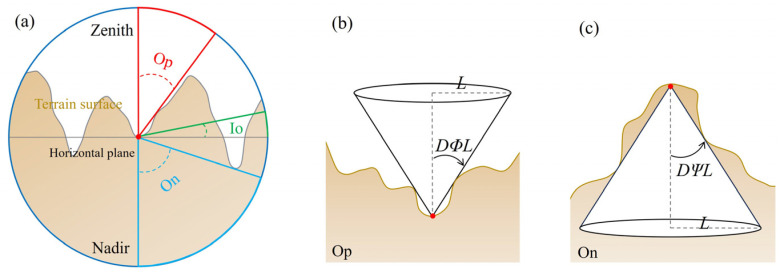
Principle of openness (modified according to Lo CM [21]).

Openness is a terrain visualization method proposed by Yokoyama et al. [38] and is widely used to display subtle surface undulations, reflecting the convex and concave changes in terrain, such as peaks, depressions, ridge lines, and valleys, by calculating the angular relationship between terrain relief and the horizontal distance within a specific range around a given point. In uneven terrain, openness is divided into positive openness (Op) and negative openness (On), representing terrain depressions and protrusions, respectively (a). Op indicates depressions in valleys or gullies (b), while On indicates protrusions on ridges or slope tops (c). The Openness map accurately reflects the three-dimensional changes in the terrain by calculating the maximum zenith and nadir angle within a radius range. It is not affected by lighting conditions and has low sensitivity to DEM noise, thus providing a more stable and precise terrain display. The principle of openness is illustrated in Figure 7. Openness values range from 0 to 1 and are influenced by HRD and SR [39]. The ridge–valley index (I), proposed by Chiba T [36], is defined as half the difference between Op and On, ranging from −1 to 1, where positive values indicate protruding terrain and negative values indicate depressed terrain.

### 4.2. Image Fusion Technology

In image editing and GIS, image fusion is a commonly used function, particularly in remote sensing image processing. Although basic image fusion methods are widely available in image editing software, such as Photoshop, the fusion capabilities in GIS and remote sensing software are relatively more limited. For example, QGIS supports several fusion modes, while ArcGIS mainly achieves simple fusion by adjusting the opacity of layers. Traditional image fusion methods typically rely on sequentially stacking multiple layers, with weaker layers placed on top and stronger layers at the bottom. However, excessive layer stacking can lead to information loss, ultimately compromising the quality of the final image.

This paper employs the RVT-py 2.2.1 tool developed by Kokalj [40], which can achieve powerful image fusion effects similar to those in Photoshop while preserving the coordinate attributes of terrain data for effective analysis. The RVT library supports six different image fusion modes, including Normal, Opacity, Screen, Multiply, Overlay, and Luminosity, and these modes can enhance the visibility of small terrain features as needed, with fusion applied sequentially from the bottom layer up. The Normal fusion mode retains the top layer and conceals the bottom layer, the Screen mode brightens the image, the Multiply mode darkens the image by multiplying brightness values, the Overlay mode enhances contrast by applying different processing methods to colors with varying brightness levels, and the Luminosity mode blends the image by retaining the luminosity of the top layer with the hue and saturation of the bottom layer, thereby altering colors without affecting shadows and textures. Additionally, the Opacity mode defines the strength of the fusion, allowing control over the visibility of the bottom layer by adjusting the opacity level. These image fusion methods offer effective solutions for enhancing the visualization of images.

### 4.3. Recognition and Extraction of Landslide Trace

#### 4.3.1. Concentration–Area Fractal Model

The C-A (Concentration–Area) fractal model is used to distinguish between geochemical background and anomalies, detect weak signals and deeply buried ore bodies, and support mineral exploration and environmental studies by analyzing the self-similarity and multi-scale characteristics of data [41]. It encompasses various fractal models such as Concentration–Area (C-A), Spectrum–Area (S-A), Concentration–Distance (C-V), Concentration–Volume (C-D), Number–Size (N-S), and multifractal singularity analysis [42]. These models help distinguish background from anomalies, enhance weak signals, and identify the distribution characteristics of mineralized zones by analyzing the spatial distribution and multiscale characteristics of geochemical data [43]. Among them, the C-A and S-A models are particularly suitable for anomaly detection in complex backgrounds.

Landslide disasters are influenced by various geological activities and exhibit complex nonlinear characteristics [44]. Following a landslide, significant terrain changes occur, leaving distinct traces on the Earth’s surface due to the sliding of surface materials, which typically manifest as tensile cracks, shear cracks, landslide scarps, landslide side walls, landslide terraces, erosion gullies, and secondary landslides. Both the traces and the overall morphology of landslides display statistical fractal characteristics, indicating their fractal nature. Therefore, the application of fractal models facilitates the identification of landslide traces. The C-A fractal model can effectively distinguish between background values and anomalous values in geological data [45]. By identifying anomalous values through this model, landslide traces can be extracted. The equation for the C-A fractal model is as follows [18]:

(3) $$A(C>v)=v^{-D}v>0,D>0$$

Taking the logarithm of both sides of the aforementioned equation yields a linear relationship between lg (c>v) and lgv. Here, lg (c>v) represents the area of regions in the image where the pixel brightness value C is greater than the set pixel brightness threshold, v is the threshold, and D denotes the fractal dimension. By plotting a scatter plot of lg (c>v) and lg v on a double logarithmic graph, a linear correlation curve is observed within a certain range. Using the least-squares method, a straight line with a slope of −*D* can be fitted. The two fitted lines represent the background area and the landslide trace area, respectively, with their intersection point being the anomaly threshold for the landslide trace. By performing an exponential operation on this intersection point, the threshold value can be determined, thereby distinguishing between the background values and the anomalous area of landslide traces. Once the anomalous values of the landslide area are obtained, this threshold can be utilized to binarize the image, dividing it into target and background areas, ultimately generating a preliminary binary map of landslide traces.

#### 4.3.2. Landslide Trace Extraction

Given the fragile geological environment in landslide areas, there may be numerous weak terrain features that result in protrusions on the terrain. After preliminary landslide traces are obtained through binarization, noise points, such as loose soil deposits, may appear. Therefore, it is necessary to denoise the extracted landslide traces. The Mean-Shift algorithm is used for denoising, as it can effectively eliminate noise while preserving the edge information of the image, thereby enhancing the accuracy of landslide trace recognition [46]. The denoised binary image is then superimposed onto the original image, and the contour extraction algorithm is utilized to extract the outer contours of the landslide traces from the binary image, ultimately achieving accurate extraction of the landslide traces through a contour drawing algorithm.

The Mean-Shift algorithm is a non-parametric clustering algorithm based on kernel density estimation, widely applied in data point clustering and pattern recognition [47]. Its core idea revolves around estimating the density distribution of data points through kernel density estimation. Kernel density estimation is a method used to estimate an unknown probability density function by placing a kernel function, such as a Gaussian kernel or uniform kernel, around each data point and aggregating the contributions of these kernel functions to estimate the overall density distribution of the data [48]. In landslide trace identification, the Mean-Shift algorithm can automatically identify high-density clusters in the landslide area through density estimation, thereby accurately locating the landslide boundary and potential landslide traces. This method is particularly suitable for complex terrain and vegetation-covered areas and can effectively distinguish landslide areas from background noise, thereby improving the accuracy of landslide identification. The formula for kernel density estimation is as follows:

(4) $$\hat{f}(x)=\frac{1}{nh^{d}}\sum_{i=1}^{n}\;K\left(\frac{x-x_{i}}{h}\right)$$

where *n* represents the number of data points, K is the kernel function, h is the bandwidth (or window width) that controls the influence range of the kernel function, and d is the dimensionality of the data. Kernel density estimation provides the Mean-Shift algorithm with an estimate of the density distribution of data points, helping the algorithm in locating areas of higher density.

Based on kernel density estimation, the Mean-Shift algorithm iteratively updates the position of each data point, causing it to gradually move towards areas of higher density and ultimately converge to local density peak points [49]. The iterative formula is as follows:

(5) $$x_{new}=\frac{\sum_{i=1}^{n}\;K\left(\frac{x\;-\;x_{i}}{h}\right)x_{i}}{\sum_{i=1}^{n}\;K\left(\frac{x\;-\;x_{i}}{h}\right)}$$

This formula can be understood as follows: the new position is the weighted average of all data points, with the weights determined by the kernel function, such that points closer to the current position are assigned greater weights. The iterative process is as follows: firstly, an initial point x is selected, then the weighted average position of $x_{new}$ under the kernel function is computed, and x is updated to the new position, $x_{new}$. This procedure is repeated until convergence is reached (i.e., the change in x is less than a certain threshold).

After multiple iterations, each data point converges to a local density peak point, which can be regarded as the center of the cluster, and all data points that converge to the same peak point are considered to belong to the same cluster. In landslide trace identification, these local peak points can effectively identify the boundaries and key feature points of the landslide area, thereby helping to accurately locate the scope and morphology of the landslide. Therefore, the Mean-Shift algorithm can complete the clustering task of the landslide area by finding all local peak points, thereby achieving efficient identification of landslide traces. The advantage of this algorithm is that it does not need to pre-specify the number of clusters, and the number of clusters is determined by the data itself, which makes it particularly suitable for processing landslide data in complex terrain, because the shape and number of landslide areas are often difficult to predict in advance. In addition, the algorithm has a good effect on irregularly shaped clusters and can effectively deal with the situation where the boundaries of the landslide area are complex and the morphology is changeable, thereby improving the accuracy and robustness of landslide identification.

## 5. Results and Discussion

### 5.1. Analysis of LiDAR-DEM Visualization Results

**Figure 8:**
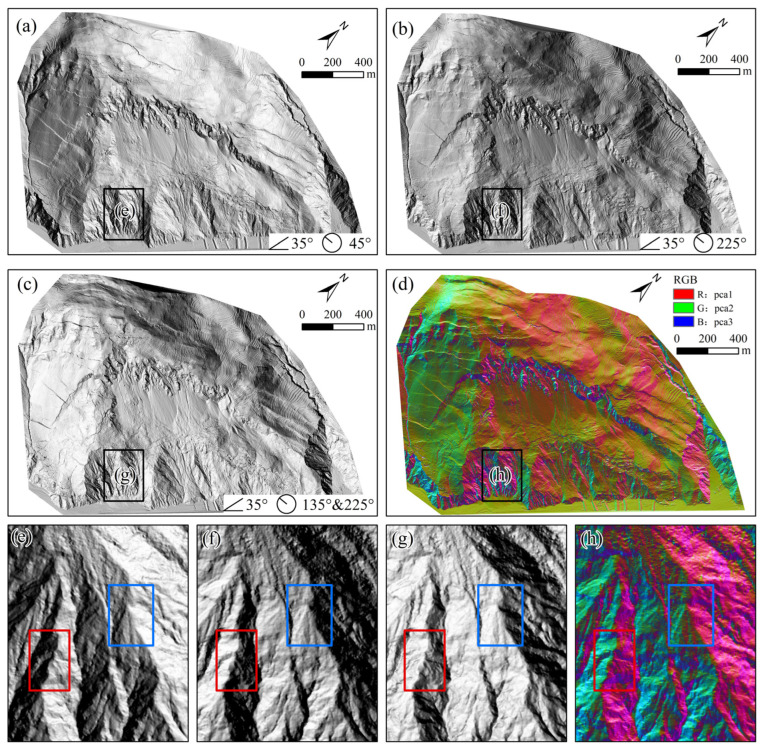
Comparative display of hillshade with different combinations: (**a**,**b**) unidirectional hillshade; (**c**) hillshade displayed through bidirectional fusion; (**d**) RGB display of multi-directional hillshade; (**e**–**h**) enlarged images corresponding to the black frames in (**a**,**b**).

Figure 8a,b display hillshade images under unidirectional solar azimuth angles of 45° and 225°, respectively. Figure 8e,f are the enlarged areas corresponding to Figure 8a,b. In the red-framed regions, there is a significant brightness difference between the two sides, with the area directly illuminated by the sun appearing brighter and the opposite area darker. The introduction of a single light source enhances the three-dimensional effect of the terrain, but it results in less distinct terrain contour features on both sides and the loss of terrain details. Therefore, a hillshade with a single light source has limitations. Figure 8c shows a composite shadow image obtained by fusing hillshade images under azimuth angles of 135° and 225°, and Figure 8g is the corresponding enlarged image. By comparing the red and blue box areas in Figure 8e–g, it is found that the overall brightness and terrain details of the composite hillshade image are improved, which alleviates the problem of detail loss caused by a single light source, indicating that fusing hillshade images from different azimuth angles can enhance terrain detail features and visual effects to a certain extent. Figure 8d is a multi-directional hillshade image with true colors, where color enhancement through principal component analysis enhances the display of terrain details. In the red box of Figure 8h, two different colors are used on both sides to emphasize terrain detail features, while in the blue box, the terrain detail features are more pronounced compared to those in the unidirectional and multi-directional fused hillshade images. The above results indicate that in the terrain visualization of hillshade, the selection and combination of light source directions need to be carefully balanced to ensure the best presentation of terrain details.

**Figure 9:**
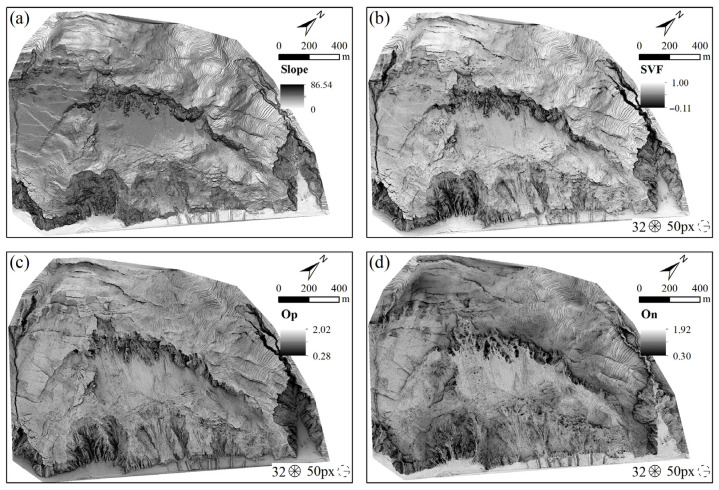
Terrain visualization maps under different visualization methods: (**a**) slope terrain visualization map; (**b**) SVF terrain visualization map; (**c**) openness terrain visualization map; (**d**) On terrain visualization map.

Terrain slope is a crucial parameter for analyzing the morphological characteristics of landslides. Analyzing the changes in slope can effectively identify the lateral boundaries of landslides. The terrain slope displayed with inverted colors can to some extent present a three-dimensional effect and is not affected by the light source, as shown in Figure 9a. In this study, the three-dimensional terrain visualization maps of SVF and openness were automatically generated using the terrain analysis tool in the digital terrain analysis software SAGA GIS 9.5.1 [50]. The main influencing parameters in the calculations were the search direction and the maximum search radius. Through referencing the relevant literature [15,51] and conducting multiple calculations, it was determined that when calculating openness and SVF using terrain data with a 0.5 m resolution in complex terrain areas, the optimal parameters were a search radius of 10 m and 16 search directions. The SVF effect map obtained based on these parameters is shown in Figure 9b, where it can be observed that the texture features are significantly enhanced compared to the hillshade and slope maps, especially in edge areas and regions with complex terrain changes. The terrain openness maps obtained according to the aforementioned parameters are presented in Figure 9c,d. Terrain openness addresses the issues of excessive shadows and over-exposure caused by parallel light sources in traditional shading rendering techniques. Although terrain openness is generally darker than SVF, it enhances the prominence of micro-terrains and better displays the concave–convex features of the terrain.

### 5.2. Optimization of Calculation Parameters for SVF and Openness

The main parameters affecting the terrain visualization effects of SVF and openness are HSD, SR, and the spatial resolution of the DEM. Given a fixed spatial resolution, since calculating SVF and openness values involves a large amount of computation, when dealing with large-scale datasets, the reasonable selection of HSD and SR is particularly critical for ensuring both computational efficiency and the quality of the results.

#### 5.2.1. Number of Horizontal Search Directions

**Figure 10:**
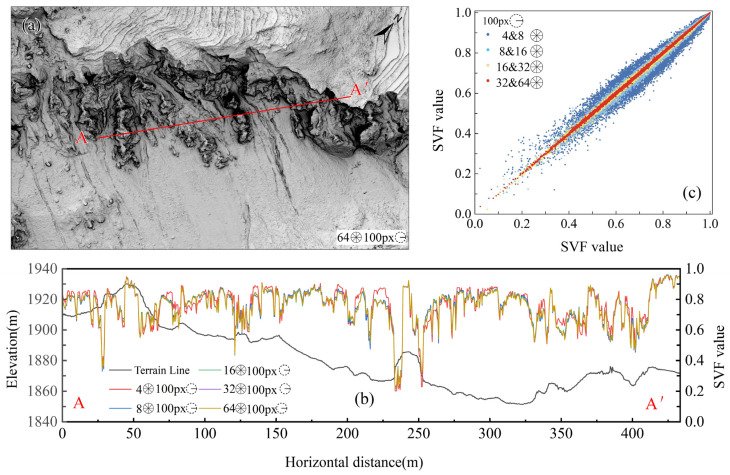
SVF visualization effects under different numbers of HSDs: (**a**) SVF images under different numbers of HSDs with SR of 100 px; (**b**) A-A’ profile under each number of HSDs; (**c**) scatter plot of SVF values under different numbers of HSDs with SR of 100 px.

Based on a DEM with a resolution of 0.5 m and a search radius of 50 m, a visualization analysis of the landslide body was conducted, evaluating the SVF for 4, 8, 16, 32, and 64 directions. The results showed that all directional settings could clearly display the characteristics of the trailing-edge cracks and minor gullies of the landslide, but as the number of directions increased, the differences in details gradually diminished. The profile (Figure 10b) indicated that the SVF value curves essentially overlapped when the number of directions exceeded 16, and the scatter plot (Figure 10c) revealed no significant differences in SVF values between 32 and 64 directions. Therefore, it is recommended to select at least 16 directions but no more than 32 directions for SVF visualization to improve computational efficiency.

#### 5.2.2. Maximum Search Radius

**Figure 11:**
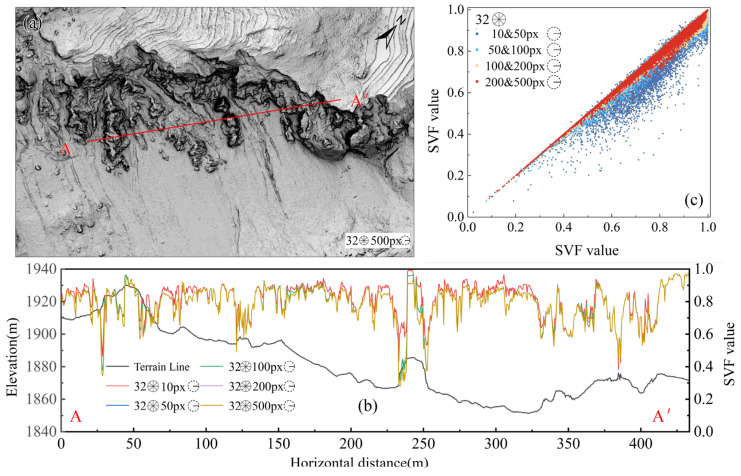
SVF visualization effect images under different SRs: (**a**) SVF images under different SRs with 32 HSDs; (**b**) A-A’ profile under each number of HSDs; (**c**) scatter plot of SVF values under different SRs with 32 HSDs.

Figure 11 presents the valley–ridge index images at an SR of 10 (5 m), 50, 100, 200, and 500 px with 32 search directions. Under a larger SR, the terrain relief features are more pronounced, but as the SR increases to a certain extent, the differences in the images gradually diminish. The profile in Figure 11b indicates that when the SR is 10 px, concave terrains are more distinctly represented. When the SR is 50 and 100 px, the profiles essentially overlap, while convex terrains become more significant at SR values of 200 and 500 px. The scatter plot in Figure 11c indicates that as the SR increases, the differences in SVF values gradually decrease; in particular, when the SR is 100, 200, and 500 px, the SVF values tend to be consistent. A radius exceeding 200 px has a limited impact on the calculations; thus, it is recommended to choose an SR of 50–200 px for areas with severe terrain relief.

Similarly, based on the comparisons between different numbers of HSDs under the same SR and different SRs under the same HSD in this study, the optimal calculation parameters for openness are determined as follows: the number of HSDs is 16 or 30, and the SR is 50–200 px.

### 5.3. Analysis of Enhanced Display Results

The purpose of enhanced display is to improve the visual effect of images and enhance the visibility of terrain features. By fusing multiple terrain visualization images, different terrain features can be highlighted. According to the requirements of landslide identification, an appropriate fusion mode is selected to achieve the desired visual effect, thereby enhancing the effectiveness and accuracy of landslide identification. Based on relevant research in the international academic community, independent enhanced display technology failed to record more than 77% of the feature information. However, by combining any three visualization techniques, over 90% of the detailed features can be resolved, significantly improving the recognizability of the information.

Slope, openness, and SVF are all terrain features that are direction-independent, meaning the highlighted terrain structures are also direction-independent. Unlike hillshade visualization techniques, these features do not take into account the effects of horizontal displacement of the terrain or human-induced modifications. Both openness and SVF visualization images enhance the display of terrain relief; Op enhances the display of small protruding terrains, such as landslide walls and landslide boundaries, while On enhances the display of recessed terrains, including cracks and erosion ditches. Although openness improves the prominence of micro-terrains, it tends to lose details of relatively flat terrains. SVF images can compensate for the detail enhancement of flat landforms, and by fusing these two types of images, the recognition of micro-terrains can be further improved.

This study employed the RVT tool to achieve the enhanced display and fusion of landslide terrain, with fusion parameters improved based on the literature [14,17], with specific settings shown in Table 2. Firstly, the hillshade image after PCA analysis was placed as the bottom layer; then, it was fused with the slope image to obtain a more three-dimensional impression; next, it was further fused with the Op and On visualization images to enhance the contrast between small terrain undulations, thereby increasing the prominence of small terrains; finally, by fusing it with SVF images, the display of flat landforms can be enhanced to a certain extent, further improving the recognizability of micro-terrains, thus obtaining the enhanced display effect of landslide terrain.

**Figure 12:**
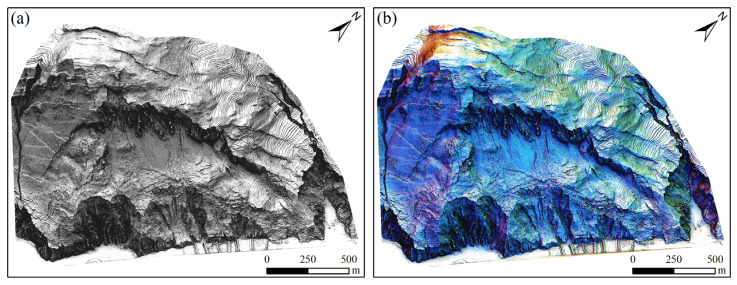
Enhanced display effect: (**a**) composite hillshade as the base map; (**b**) RGB display of multi-directional hillshades as the base map.

The fused image generated through this composite method, as illustrated in Figure 12, intuitively displays the topographic features and texture details of the landslide terrain, demonstrating a more prominent effect compared to single visualization techniques. In particular, it shows remarkable performance in enhancing color differentiation and texture features, effectively improving the visual recognition of the landslide. Furthermore, the images enhanced by the image fusion technology effectively reveal the distribution characteristics of landslide elements, providing a more precise and intuitive scientific basis for landslide risk assessment and disaster prevention and mitigation.

### 5.4. Landslide Trace Recognition and Extraction

#### 5.4.1. Coarse Identification of Landslide Traces Based on the C-A Fractal Model

**Figure 13:**
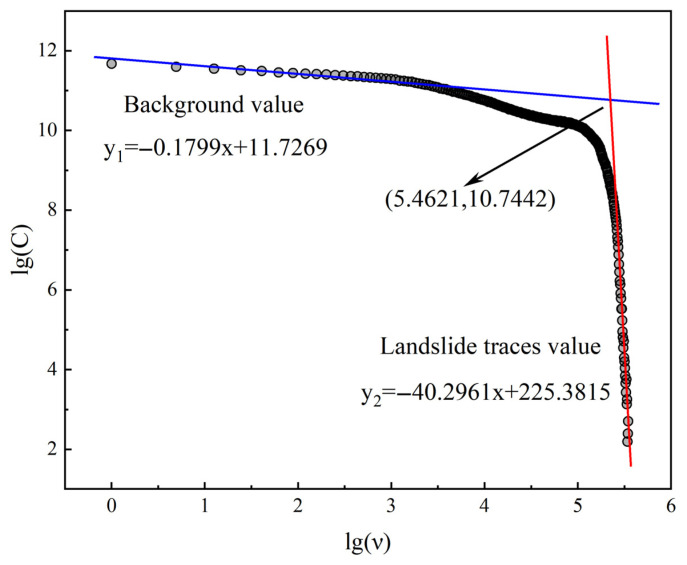
Fractal double-logarithmic diagram of the enhanced display image in the study area.

The process of using the C-A fractal model to obtain landslide traces and outliers involves the following steps: Firstly, a statistical analysis of the gray values in the image is conducted. In addition, a double-logarithmic scatter plot using the gray data of the image is generated, with respect to the variables lgA(C>v) and lgA(v). The scatter plot is then fitted using the least-squares method to obtain two lines with different slopes. Finally, an exponential calculation on the intersection point of these two lines is used to determine the anomaly threshold for landslide traces. The C-A fractal double-logarithmic plot obtained from the statistical analysis of the gray values in the image is shown in Figure 13.

By performing linear fitting on the discrete points generated from the grayscale data using the least-squares method, two fitting lines with different slopes can be obtained. These two lines represent the background values of non-landslide traces and the values of landslide traces within the study area, respectively. The y_1_ line has a smaller slope, representing the background value of non-landslide traces. The coefficient of determination of this line is R^2^ = 0.973, indicating that the model can explain 97.3% of the data variation; the RMSE is 0.032, and the MAE is 0.025, showing that the fitting has a high precision. The y_2_ line, whose slope is significantly smaller than y_1_, represents the terrain anomaly caused by the landslide. Those outliers are regarded as discrete values, indicating the landslide traces formed after the landslide event. The coefficient of determination of this line is R^2^ = 0.985, indicating that the model can explain 98.5% of the data variation; the RMSE is 0.031, and the MAE is 0.023, also showing that the fitting has a high precision. The intersection point of the two fitting lines is the sought threshold for determining the discrete values of landslide traces. By solving for the intersection point, the outliers can be identified. The x-coordinate value of the intersection point of the two fitting lines is 5.4621. Through exponential calculation based on this value, the lower limit of the abnormal grayscale value in the enhanced display image is determined to be 10.7442. In other words, when the grayscale value of an image pixel reaches 107.442, it indicates the presence of landslide traces.

**Figure 14:**
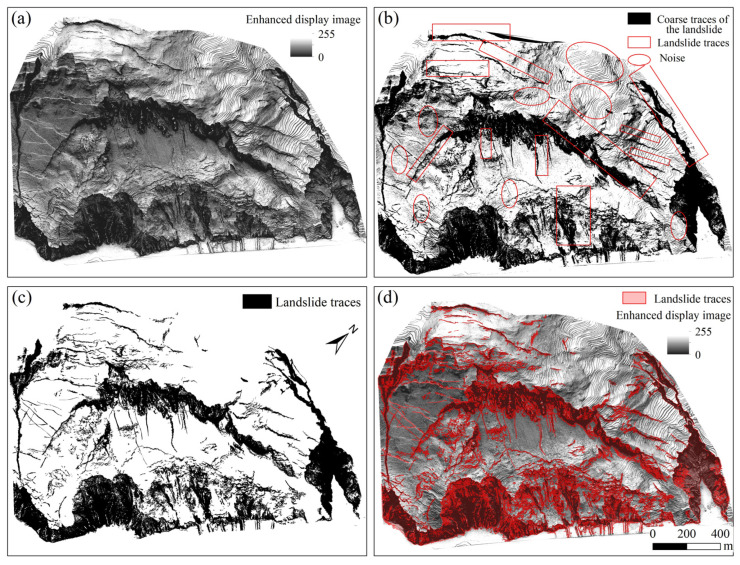
Landslide trace extraction map: (**a**) enhanced display image; (**b**) rough extraction result of landslide traces; (**c**) denoised result of landslide trajectories; (**d**) enhanced display image with landslide traces overlaid.

By utilizing the threshold obtained from the fractal model and performing binarization on the enhanced display image based on the OpenCV platform, rough landslide traces within the study area can be obtained, as shown in Figure 14b. In the image, pixels exceeding the threshold represent landslide traces, while those below the threshold indicate background areas. The black regions in the image correspond to landslide traces, and the white regions represent the background. Figure 14a displays the landslide area and its features such as cracks, landslide walls, and erosion gullies, enabling effective identification of landslide traces. However, due to the fragile geological environment in the landslide-prone area, small soil clods with weak undulations may accumulate and form noise after binarization. Therefore, to extract landslide traces more accurately, it is necessary to further denoise the extraction results, remove the noise, and extract the landslide traces.

#### 5.4.2. Landslide Trace Denoising and Extraction

To further improve the accuracy of landslide trace recognition, this study employed the Mean-Shift algorithm based on the OpenCV platform to denoise the extracted landslide features, with the image before denoising shown in Figure 14b. The Mean-Shift algorithm is a non-parametric density-based clustering method that defines clusters by locating regions with the highest density of data points, without the need to specify the number of clusters in advance. Its main steps are as follows: first, determining an appropriate bandwidth value as the influence range for each data point, then using a Gaussian kernel function to calculate the weighted average position of all points within the neighborhood of each data point, and moving the data point to that position, repeating this process until convergence. Ultimately, data points with the same or very similar final positions are grouped into the same cluster, achieving denoising and clustering of the dataset. Through denoising, noise and irrelevant interference caused by weak terrain protrusions can be effectively eliminated, while retaining the authentic information of landslide traces, thereby further improving the accuracy of landslide trace extraction. The denoised images are presented in Figure 14c,d.

**Figure 15:**
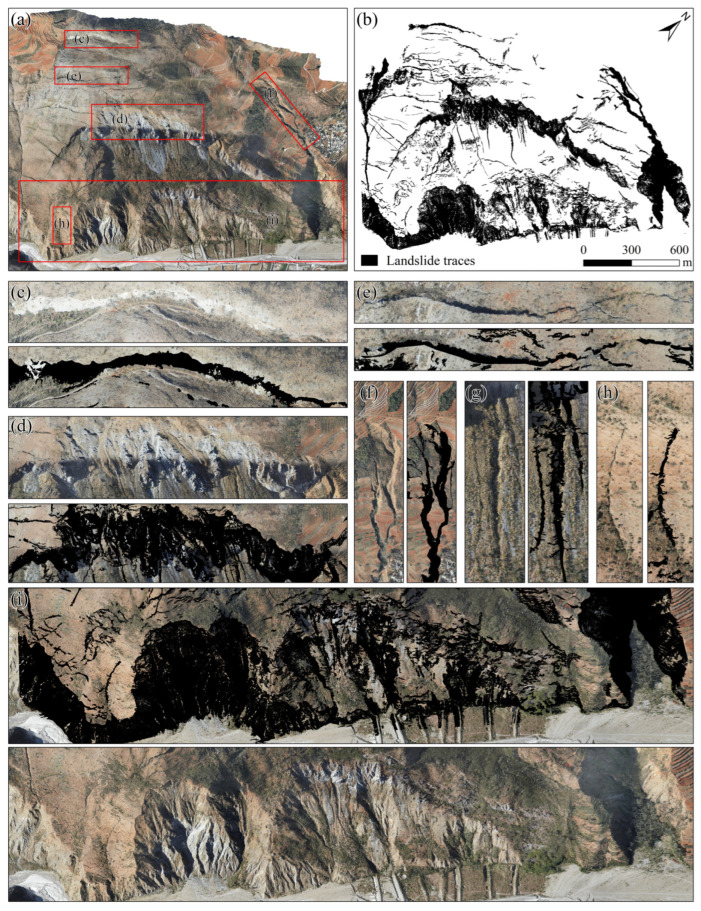
Comparison of landslide traces: (**a**) UAV orthophoto; (**b**) extracted landslide traces; (**c**,**e**) comparison images of the extracted shear cracks at the rear edge of the landslide; (**d**) comparison image of the extracted rear wall of the landslide; (**f**–**h**) comparison images of the extracted erosion gullies on the landslide body; (**i**) comparison image of the extracted erosion area at the front edge of the landslide.

This method effectively extracted landslide traces, and in the final results, cracks, steps, and subsidence areas around and within the landslide were successfully identified. To validate the effectiveness of the identification results in this study, an orthophoto of the landslide area was constructed using UAV images acquired during the same period (Figure 15a). By comparing the images in Figure 15, it can be observed that the influence of vegetation can be effectively eliminated from the LiDAR-DEM enhanced display image, allowing for the extraction of cracks at the rear edge of the landslide (Figure 15c,e), landslide walls (Figure 15d), erosion gullies (Figure 15f–h), and secondary landslides and severely eroded areas at the front edge (Figure 15i). The comparison between the orthophoto and the trace extraction results indicates that the identification and extraction of the traces were both effective.

### 5.5. Landslide Trace Extraction Comparison

This paper proposes a method for the enhanced display of landslide terrain and extraction of landslide trajectories based on a LiDAR-DEM. The purpose is to enhance the identification of landslide traces and facilitate a deeper understanding of the topographic and geomorphic characteristics of landslides. This section will discuss the proposed method and explore the advantages of its application. First, the point cloud data obtained by airborne LiDAR is used as the data source to construct a high-precision DEM to generate images such as hillshade, slope, openness, and SVF. Then, the pixel-based image fusion method is used to obtain the enhanced display image to effectively enhance the micro-topographic characteristics of the landslide micro-topography. Finally, based on fractal theory, landslide traces are extracted from the enhanced display image. By applying fractal theory to segment the image and using the Mean-Shift algorithm to denoise, the landslide traces can be accurately located and extracted, and the accuracy of landslide trace identification can be improved. Compared with traditional mathematical analysis methods, fractal theory is based on the self-similarity between objects and can reduce human intervention.

Compared with optical remote sensing, the landslide trajectory identification method using airborne LiDAR data has significant advantages. Optical remote sensing mainly relies on two-dimensional images and has difficulty obtaining terrain information, while airborne LiDAR can obtain three-dimensional point cloud data and provide more comprehensive terrain details. By constructing a high-precision DEM and generating enhanced display images, the landslide terrain characteristics can be comprehensively displayed from multiple angles, thereby realizing the accurate identification of landslide traces.

In order to verify the reliability of the fractal model threshold segmentation method in this paper, this study introduced the classic OSTU threshold segmentation method as a comparison. OSTU determines the optimal segmentation threshold by maximizing the inter-class variance of the image foreground and background and is widely used due to its high efficiency [52]. This study compares OSTU with the method in this paper on the same dataset and evaluates the superiority of the method in this paper in terms of segmentation accuracy, noise resistance, and adaptability to complex terrain and vegetation-covered areas.

**Figure 16:**
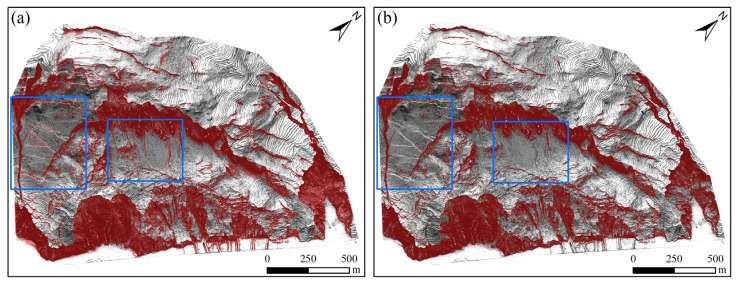
Comparison of landslide trace extraction using different threshold segmentation methods. (**a**) the fractal model threshold segmentation method used in this study; (**b**) the OSTU threshold segmentation method.

The recognition results (Figure 16) show that in the comparative experiment, it can be clearly seen by observing the pictures that the segmentation effect of the OSTU method on the right picture is not as good as the fractal model threshold segmentation method proposed in this paper. Specifically, when dealing with complex terrain and vegetation-covered areas, the OSTU method has low segmentation accuracy and cannot accurately distinguish the landslide area from the background, resulting in some landslide features being missed or misjudged. In the two blue boxes in this figure, the method of this paper can better extract landslide traces, while the OSTU method has a poor segmentation effect due to the influence of terrain. In contrast, the method of this paper can capture the details of the landslide area more finely, its segmentation results are clearer and more accurate, and it has stronger adaptability to complex environments. It can effectively avoid mis-segmentation and missed segmentation, thereby better meeting the actual needs of landslide identification.

In summary, all the fractal model methods in this paper can better obtain thresholds by taking double logarithms and segment images to extract landslide traces. However, the OSTU method cannot accurately distinguish the landslide area from the background in areas with complex terrain, resulting in some landslide features being missed or misjudged.

## 6. Conclusions

In this study, the application of airborne LiDAR technology combined with terrain visualization technology in enhanced landslide display and trace recognition was explored. Firstly, a high-precision LiDAR-DEM was generated using airborne LiDAR point cloud data; secondly, terrain visualization images of the DEM were created using hillshade, slope, SVF, and openness techniques; then, an enhanced display image was obtained through a pixel-based image fusion method to effectively enhance the significance of the micro-terrain features of the landslide. Finally, based on the fractal characteristics of the enhanced display image, a fractal model was employed to obtain a threshold, enabling the precise extraction of landslide traces. The following conclusions were drawn:

(1) Enhanced display of landslide terrain based on LiDAR-DEM. Firstly, visualization images are generated using slope, SVF, openness, and hillshade techniques, and then pixel-level image fusion methods are applied to integrate the features of different visualization images, resulting in an enhanced display image of landslides. This image enhances the capability to identify typical landslide geomorphic features such as cracks at the rear edge, landslide walls, erosion gullies, landslide steps, and erosion areas at the front edge, facilitating the accurate extraction of landslide information.
(2) Landslide trace extraction based on enhanced display images. By utilizing the image value characteristics of landslide and non-landslide areas in the enhanced display images, a threshold is obtained through a fractal model for image segmentation. Subsequently, the Mean-Shift algorithm is employed for denoising, which enables the effective extraction of landslide traces and achieves semi-automated landslide trace extraction, overcoming the limitations of traditional methods that rely on manual threshold selection for landslide trace recognition.
(3) Framework for enhanced landslide terrain display and trace recognition. This paper presents a method for enhanced landslide terrain display and trace recognition based on airborne LiDAR data, integrating various terrain visualization techniques and image fusion technologies to achieve enhanced display of landslide terrain and integrating fractal models with denoising algorithms for trace recognition and extraction. This framework optimizes the presentation of terrain enhancement visualization features and trace recognition in landslide-prone areas, enhancing the accuracy and efficiency of landslide trace recognition through the application of multiple integrated technologies.

In this study, we used airborne LiDAR data to enhance the display of landslide terrain, determined the threshold through a fractal model, and then applied the Mean-Shift algorithm for denoising and the identification and extraction of landslide traces. However, due to the complexity and high hazard of landslide terrain, field measurements cannot fully cover all areas, which limits our ability to conduct complete digital quantitative analysis of landslide traces, thereby affecting the integrity of accuracy and error assessment. In addition, this study only took a single landslide as the research object, and due to funding constraints, it was not possible to obtain airborne LiDAR data of more landslides to verify the universality of the proposed model. Despite these limitations, this study still provides a preliminary analytical framework and method for landslide identification and trace extraction. In future studies, we will combine UAV mapping and high-resolution remote sensing images to conduct more comprehensive field surveys and collection of landslide data to overcome the shortcomings of current research.

## Figures and Tables

**Table 1:** Specifications of the DJI Zenmuse L2 airborne LiDAR system and flight parameters.

Equipment	Metric	Parameter
LiDAR System	Range/m	450
	Range Accuracy/cm	±2
	Number of Returns	5
	Laser Wavelength/nm	905
POS	Horizontal Accuracy/cm	1
	Vertical Accuracy/cm	1.5
Mapping Camera	Effective Focal Length/mm	2000
	Focal Length/nm	24
Flight Parameters	Relative Flight Altitude/m	180
	Side Overlap/%	30
	Flight Speed/(m/s)	10
	Laser Pulse Rate/kHz	240

**Table 2:** Fusion parameters for enhanced display image layers.

Image Type	Calculation Parameter Settings	Color Gradient	Fusion Method	Fusion Order	Opacity
SVF	Search radius: 5, number of search directions: 16	Black to White	Multiply	4	25%
Op	Search radius: 5, number of search directions: 16	Black to White	Overlay	3	50%
On	Search radius: 5, number of search directions: 16	White to Black	Overlay	2	50%
Slope		Black to White	Luminosity	1	50%
Hillshade	SEA: 35°, SAA: 45°, 135°, and 225°	Black to White	Opacity	0	100%
	RGB-PCA-Hillshade	RGB			

## Data Availability

The data presented in this study are available from the corresponding author on request.
